# Isobaric Tags for Relative and Absolute Quantitation (iTRAQ)-Based Proteomic Analysis of Hugan Qingzhi and Its Protective Properties against Free Fatty Acid-Induced L02 Hepatocyte Injury

**DOI:** 10.3389/fphar.2017.00099

**Published:** 2017-02-28

**Authors:** Fan Xia, Xiaorui Yao, Waijiao Tang, Chunxin Xiao, Miaoting Yang, Benjie Zhou

**Affiliations:** Center for Drug Research and Development, Zhujiang Hospital, Southern Medical UniversityGuangzhou, China

**Keywords:** Hugan Qingzhi (HQT), non-alcoholic fatty liver disease, isobaric tags for relative and absolute quantitation (iTRAQ), proteomics, hepatocyte, non-alcoholic fatty liver disease (NAFLD)

## Abstract

In previous research, Hugan Qingzhi, a traditional Chinese medicine, was shown to have protective effects against hepatic steatosis. However, its activity against non-alcoholic fatty liver disease (NAFLD) and the mechanisms by which it exerts its effects remain unknown. In the present study, the effects of Hugan Qingzhi on free fatty acid (FFA)-induced L02 cells were examined. The techniques of iTRAQ labeling, together with strong cation exchange-non-liquid chromatography–tandem mass spectrometry (SCX-non-LC-MS/MS) analysis and serum pharmacology, were used to evaluate the effects of Hugan Qingzhi-medicated serum on FFA-induced L02 hepatocyte injury. Results identified 355 differentially expressed proteins following FFA treatment, compared with a control group; 359 altered proteins in the Hugan Qingzhi high dose + FFA treatment group, compared with the FFA treatment group; and 365 altered proteins in the Hugan Qingzhi high dose + FFA treatment group, compared with the control group. Based on the Kyoto Encyclopedia of Gene and Genomes pathway enrichment analysis, it is concluded that several pathways including those of microbial metabolism in diverse environments, fatty acid metabolism, peroxisome proliferator activated receptor signaling, and mitogen-activated protein kinase signaling are closely associated with the effects of Hugan Qingzhi-medicated serum in FFA-induced L02 hepatocyte injury. Furthermore, several differentially expressed proteins, including heat shock protein 27 (HSP27), acetyl-CoA acetyltransferase 1, calnexin, and integrin-linked kinase, were validated by western blotting. A target-specific HSP27 siRNA was used to investigate further the function of HSP27, and it was found that HSP27 might have a key role in the observable effects of Hugan Qingzhi-medicated serum in FFA-induced L02 hepatocyte injury. The results not only confirmed that Hugan Qingzhi exhibits a significant protective effect in FFA-induced L02 hepatocyte injury, but also suggest insights into the mechanism of such protective effects.

## Introduction

Non-alcoholic fatty liver disease (NAFLD) is defined as the presence of hepatic steatosis in the absence of any secondary causes of hepatic accumulation, such as significant alcohol consumption, steatogenic drugs, or hereditary disorders. Clinically, metabolic syndrome and its many components such as obesity, diabetes mellitus, and dyslipidemia (Angulo, 2002) are closely associated with NAFLD. As a clinicopathologic syndrome, NAFLD includes a spectrum of liver disorders, ranging from steatosis to steatohepatitis and fibrosis (Sheth et al., 1997; Brunt et al., 1999). Non-alcoholic steatohepatitis (NASH) is a more aggressive condition within the spectrum of NAFLD, characterized by steatosis, inflammation, and hepatocyte injury (ballooning degeneration) with or without cirrhosis (Sheth et al., 1997; Brunt et al., 1999; Angulo, 2002). Although many risk factors are known to play a pivotal role in the development of NAFLD, its pathogenesis and progression remain poorly understood (Farrell, 2002; Guturu and Duchini, 2012). Thus, it is necessary to elucidate the overall processes and signaling pathways of NAFLD and the mechanisms associated with its treatment.

Within recent years, proteomics has been widely used in the life sciences to detect differentially expressed proteins and novel targets, and discover the associated mechanisms of action (Lai et al., 2015; Pu et al., 2015; Wei et al., 2016). Isobaric Tags for Relative and Absolute Quantification (iTRAQ) is a novel technique developed by Applied Biosystems Incorporation (ABI) with many advantages, such as high throughput, high sensitivity, and superior accuracy, and has grown to be a robust method in comparative proteomics (Treumann and Thiede, 2010).

Hugan Qingzhi (HQT) is a traditional Chinese medicine used for treatment of NAFLD. Its therapeutic effects on NAFLD have been confirmed in previous studies (Yin et al., 2014; Tang et al., 2015). It consists mainly of five plant materials: including Rhizoma Alismatis, Fructus Crataegi, Pollen Typhae, Folium Nelumbinis, and Radix Notoginseng (Yin et al., 2014). However, its complex composition poses considerable challenges for studies on the mechanisms of HQT, because the effect of direct addition of a crude drug to an *in vitro* system does not reflect the actual efficacy of the drug (Yin et al., 2014). To minimize interference between the physical and chemical characteristics of crude drugs in an *in vitro* study, the experimental conditions should be as similar as possible to those of the *in vivo* environment (Bochu et al., 2005; Meng et al., 2012; Yin et al., 2014). Thus, serum pharmacology coupled with iTRAQ were used in the present study to determine the protective effect of HQT in a free fatty acid (FFA)-induced L02 hepatocyte injury model.

## Materials and Methods

### Plant Material Extract and Preparation of HQT

Preparation of HQT has been described in previous studies (Yin et al., 2014). The method entailed boiling a mixture of 30% Rhizoma Alismatis, 30% Fructus Crataegi, 15% Pollen Typhae, and 20% Folium Nelumbinis, followed by refluxing in 70% ethanol (6:1, v/w) for 2 h. The ethanol extract was then collected. The residue was filtered and again extracted by the aforementioned method. The ethanol extracts were pooled and evaporated to dryness under reduced pressure. The yield of the original dried mixture was 14.45% (w/w). Radix Notoginseng (5%) was then ground, sifted, and added to the dried extract to produce HQT. The basic quality of HQT was checked by thin-layer chromatography and high-performance liquid chromatography (HPLC) analyses (Zhou et al., 2012).

### Preparation of HQT-Medicated Serum

A vehicle control group, three groups of different doses of HQT-medicated serum (low, moderate, and high), and fenofibrate-medicated serum (FF) were produced according to methods described in a previous study (Yin et al., 2014). Thirty male Sprague Dawley rats (weighing 200 ± 20 g), purchased from the Animal Experiment Center of Southern Medical University, Guangzhou, China, were maintained on a regular 12-h light/dark cycle at 25–28°C for a week. The rats were randomly divided into five groups and orally administered different doses of HQT, fenofibrate, or 1 mL/100 g of saline for 7 days (**Table 1**) (Yin et al., 2014). One hour after the final treatment, blood was collected aseptically via the abdominal aorta and centrifuged. The serum from the same group was then pooled, filtered via 0.22 μm filters, inactivated at 56°C for 30 min, and stored at -80°C. Results of HPLC analysis of HQT-medicated serum were reported in a previous study (Yin et al., 2014). All animal experiments were approved by the Southern Medical University Animal Ethics Committee and carried out in accordance with the institutional guidelines.

**Table 1:** Data on medicated serum preparation for each experimental group.

Group information of medicated serum	Dosage
Vehicle control group	1 mL/100 g of saline/day
Low dose of HQT medicated serum group	2.7 g/kg/day
Moderate dose of HQT medicated serum group	5.4 g/kg/day
High dose of HQT medicated serum group	10.8 g/kg/day
Fenofibrate-medicated serum group	0.4 g/kg/day

### Cell Culture and Experiment Design

Human hepatocyte cell line L02 was obtained from China Cell Culture Center (Shanghai, China) and cultured in RPMI-1640 Medium (Gibco, USA), containing 10% fetal bovine serum (Gibco, USA) at 37°C, under 5% CO_2_. The cells were divided into six groups as follows: the control group; FFA (oleic acid : palmitic acid at 2:1) group; FFA + 10% low dose HQT-medicated serum group (HL); FFA + 10% moderate dose HQT-medicated serum group (HM); FFA + 10% high dose HQT-medicated serum group (HH); and FFA + 10% fenofibrate-medicated serum (FF). To verify whether the differences observed with the HQT-medicated serum were attributable to HQT rather than the serum, 10% vehicle serum control was added to the control and FFA groups. The final concentration of FFA in each group was 1 mM.

### LDH Assay of Each Medicated Serum on L02 Cells

According to a previously described method (Yin et al., 2014), the cytotoxicity of each medicated serum on L02 cells was evaluated by the lactate dehydrogenase (LDH) release assay. Briefly, cells were cultured with 10% vehicle serum, 10% low, moderate, or high dose HQT-medicated serum, or 10% fenofibrate-medicated serum for 24 h. LDH release in the supernatant was detected using a colorimetric assay kit according to the manufacturer’s instructions (Nanjing Jiancheng Bioengineering Institute, Nanjing, China).

### Biochemical Assay

To estimate the protective effects of HQT in FFA-induced L02 cell injury, several biochemical parameters, such as triglycerides (TG), aspartate aminotransferase (AST), alanine aminotransferase (ALT), malondialdehyde (MDA), super-oxidase dismutase (SOD), and glutathione (GSH) were measured using a colorimetric assay kit from Nanjing Jiancheng Bioengineering Institute (Nanjing, China). L02 cells were cultured in a 6-well plate at a density of 1 × 10^5^ per well and allowed to grow to the desired confluence. The cells were then treated for 24 h with Gibco RPMI-1640 containing various concentrations of HQT-medicated serum (HL, HM, and HH) or fenofibrate-medicated serum (FF), before stimulation with 1 mM FFA for 24 h.

### Analysis of ROS Generation

Levels of intracellular reactive oxygen species (ROS) in L02 cells were measured quantitatively and qualitatively using 2′,7′-dichlorodihydrofluorescein diacetate (DCF-DA), a non-polar dye that can diffuse into cells and then hydrolyze to non-fluorescent 2′,7′-dichlorofluorescein (DCFH). ROS can oxidize cellular DCFH to highly fluorescent dichlorofluorescein (DCF), thus levels of intracellular ROS can be measured by the intensity of fluorescence of DCF. In the present study, cells were seeded in 6-well plates at a density of 1 × 10^5^/well. The cells were treated for 24 h with Gibco RPMI-1640 containing HQT-medicated serum (HL, HM, and HH), or fenofibrate-medicated serum (FF), before stimulation with 1 mM FFA for 2 h, and incubation for 30 min with DCFH-DA (10 μM) at 37°C. Levels of intracellular ROS were determined by measuring the fluorescence density of DCF using a SpectraMax M5 multifunctional microplate reader (Molecular Devices, Shanghai, China).

### Oil Red O Staining

Oil Red O staining was performed to facilitate lipid visualization and quantification. L02 cells were fixed with 4% paraformaldehyde and stained with 0.7% Oil Red O solution for 30 min at 25°C. Cells were then washed and counterstained with hematoxylin for 5 min. Images were acquired using the Olympus BX51 image system (Olympus, Tokyo, Japan).

### Inflammatory Cytokine Measurements

Expression of IL-6 and TNF-α were analyzed using ELISA kits (MultiSciences, Hangzhou, China) according to the manufacturer’s instructions. Absorbance was read at 450 nm using a SpectraMax M5 multifunctional microplate reader (Molecular Devices, Shanghai, China).

### Transfection of Small Interference RNA

Human HSP27-specific siRNA and siRNA-control were designed and synthesized by RiboBio Inc. (Guangzhou, China) for transfection. The sense and antisense strands of HSP27-specific siRNA were 5′-AUGAGACUGCCGCCAAGUAdTdT-3′ and 5′-UACUUGGCGGCAGUCUCAUdTdT-3′, respectively. Cells were plated in 6-well plates at 30–50% confluence, then transfected with 50 nM siRNA-targeting HSP27 or 50 nM siRNA-control using Lipofectamine 2000 (Life Science, CA, USA). After 6 h, 1 mL of Gibco RPMI-1640 containing 10% fetal bovine serum was added to each well. Cells were transfected with siRNA for 24 h and then treated with, or without FFA and HQT-medicated serum.

### Cell Harvest and Protein Sample Preparation

L02 cells were washed twice with ice-cold PBS and harvested by scraping in cold PBS after the various treatments for each group (control, FFA, and HH). After centrifugation at 1000 × *g* for 10 min at 4°C, cell pellets were collected and lysed in buffer containing 8 M urea, 2 M thiourea, 4% CHAPS, 5% DNA enzyme, and 5% RNA enzyme. The lysates were then intermittently sonicated for 30 s. The resulting lysate was centrifuged at 12,000 × *g* for 1 h at 4°C, and the supernatant was collected. Total protein concentration was measured using the Bradford method. All samples were stored at -80°C until further analysis.

### iTRAQ Labeling

Proteins were precipitated following the addition of a fourfold volume of cold acetone containing 10 mM DTT for about 2 h at -20°C, and centrifugation at 12,000 × *g* for 20 min at 4°C, followed by air-drying and dissolution with 100 μL TEAB dissolution buffer. The Bradford method was used to quantify each protein sample. From each sample, 100 μg of protein was reduced, alkylated, and then digested by Trypsin (Promega, Madison, WI, USA) at 37°C for 16 h, with the ratio of protein : trypsin = 20:1, and labeled using the iTRAQ Reagent-8 plex Multiplex Kit (SCIEX, Framingham, MA, USA) according to the manufacturer’s protocol. Two biological replicates were carried out for the control group, and three biological replicates for the FFA and HH groups. The control group samples were labeled with 113 and 114; FFA group samples were labeled with 115, 116, and 117; HH group samples were labeled with 118, 119, and 121.

### Strong Cation Exchange (SCX) Chromatography

The iTRAQ labeled peptides were mixed in equal amounts and fractioned by an HPLC system (Thermo Scientific Dionex UltiMate 3000 BioRS, Thermo Scientific, Shanghai, China) using the Durashell C18 column (5 μm, 100 Å, 4.6 mm × 250 mm). The dried peptides were dissolved in 100 μL buffer A (20 mM NH_4_HCO_3_ in 3% acetonitrile, pH 10), and were then loaded onto the HPLC device at a flow rate of 1 mL/min with buffer B (20 mM NH_4_HCO_3_, pH 10) at gradients of 0–8% for 5 min; 8–45% for 25 min; 45–80% for 15 min; and 80–100% for 5 min. The eluted fractions were collected at 1-min intervals and pooled into 12 fractions, then desalted with a Strata X C18 column (Phenomenex, Guangzhou, China) and vacuum-dried.

### LC-MS/MS Analysis

Liquid chromatography-electrospray ionization-tandem mass spectrometry (LC-ESI-MS/MS) analysis was performed on the AB Sciex non-LC-MS/MS (Triple TOF 5600 plus) system. Peptides were loaded onto a 20 μm PicoFrit emitter (New Objective) packed to 12 cm with Magic C18 AQ beads (3 μm, 120 Å) for the stationary phase. Chromatography was performed with a 90-min gradient from 2 to 30% [buffer A: 0.1% (v/v) formic acid and 5% (v/v) acetonitrile; buffer B: 0.1% (v/v) formic acid and 95% (v/v) acetonitrile]. MS1 spectra were collected in the range 350–1500 m/z for 250 ms. The 20 most intense precursors with charge states 2–5 were selected for fragmentation. MS2 spectra were collected in the range 50–2000 m/z for 100 ms. Precursor ions were excluded from reselection for 15 s.

### Data Processing and Analysis

The original MS/MS file data were submitted to the ProteinPilot Software v 4.5 for analysis. The Paragon algorithm (Shilov et al., 2007) was integrated into ProteinPilot against UniProt human database (142483 items, updated April, 2015) for database searching and protein identification. The parameters were set as follows: the instrument was TripleTOF 5600, for iTRAQ quantification, and cysteine was modified with iodoacetamide; biological modifications included ID focus and trypsin digestion. The Quantitate, Bias Correction, and Background Correction were checked for protein quantification and normalization. The resulting 1% global fits from FDR corresponding to 99% correct protein identification was used as an initial qualification criterion. In addition, only proteins with at least one unique peptide and unused protein score of >1.3 were considered for further analysis. Some of the peptides identified were excluded from the quantitative analysis for one of the following reasons: (i) peaks corresponding to the iTRAQ labels were not detected; (ii) peptides were identified with low identification confidence; (iii) peptides were claimed by more than one protein; (iv) S/N (signal-to-noise ratio) for any peptide ratio was too low; (v) combined feature probability <30%, such as semi-tryptic peptides, peptides missing an iTRAQ reagent label, and those with low probability modifications, or large delta masses. For biological replicates or technological replicates, the average fold change induced by treatment relative to the control or FFA group was defined. Statistically significant differences between expression levels of the proteins in various samples were determined by the Student’s *t*-test (two-tailed or unpaired), and the minimum values among replicates were treated as the final *P*-values. Data were expressed as mean ± SD. For protein abundance ratios measured with iTRAQ, we took fold changes ≥1.2 (or ≤0.83) and *P*-values < 0.05 were taken as the thresholds to identify significant changes.

### Bioinformatic Analysis of Proteins

For bioinformatic analyses, differentially expressed proteins were classified based on annotations from the UniProt Knowledgebase^1^. GO functional classification was performed with the Blast2GO software (v4.5 pipeline5). The KEGG databases^2^ were used to access current knowledge on biochemical pathways and other types of molecular interactions. Both GO and KEGG pathway enrichment analyses were used to uncover the enriched pathways of significantly altered proteins and to identify their functions.

### Immunoblotting Analysis

Cell culture medium was removed from each well, rinsed twice with PBS, and lysed with EnoGene^TM^ Total Protein Extraction Kit (KeyGEN Biotechnology, Nanjing, China), according to the manufacturer’s instructions. Total protein concentrations were determined by the Bradford method, and equal amounts of proteins from different groups were separated via 12 or 15% sodium dodecyl sulfate polyacrylamide gel electrophoresis (SDS-PAGE) following standard protocols. Primary antibodies including anti-human polyclonal antibodies that recognize ACAT1, CANX, ILK, and GAPDH purchased from Proteinteach Group (Wuhan, China); rabbit anti-human phospho-HSP27 (Ser15) antibody and HSP27 antibody purchased from Affinity Bioscience (USA); and secondary antibodies, goat anti-rabbit horseradish peroxidase (HRP)-IgG purchased from Zhongshan Golden Bridge Biotechnology Co, Ltd. (Beijing, China) were used in the present study.

### Statistical Analysis

Statistical significance was analyzed using the Student’s *t*-test (paired or unpaired) or one-way analysis of variance (ANOVA) with the GraphPad Prism software (version, 5.0). Data are presented as mean ± standard deviation (SD). Differences were considered to be statistically significant at *p* < 0.05.

## Results

### Cytotoxic Effects of Each Medicated Serum on L02 Cells

First, LDH release in supernatant was measured to evaluate the cytotoxic effects of each medicated serum on L02 cells. The results showed that 10% vehicle serum, 10% HQT-medicated serum (low, moderate, high), and 10% fenofibrate-medicated serum treatments have no significant difference on LDH release (*p* > 0.05), compared with the vehicle control group (**Table 2**), indicating that each medicated serum treatment has no cytotoxic effects on L02 cells.

**Table 2:** Lactate dehydrogenase (LDH) release assay for each medicated serum treatment.

Group information of medicated serum treatment	LDH release (U/L)
Vehicle control group	102.45 ± 14.02
10% vehicle serum group	108.01 ± 10.21^Δ^
10% Low dose of HQT medicated serum group	109.86 ± 14.43^Δ^
10% Moderate dose of HQT medicated serum group	111.70 ± 10.98^Δ^
10% High dose of HQT medicated serum group	112.74 ± 11.86^Δ^
10% Fenofibrate-medicated serum group	108.97 ± 15.36^Δ^

*^Δ^*p* > 0.05.*

### Effects of HQT-Medicated Serum in FFA-Induced LO2 Cells

**FIGURE 1:**
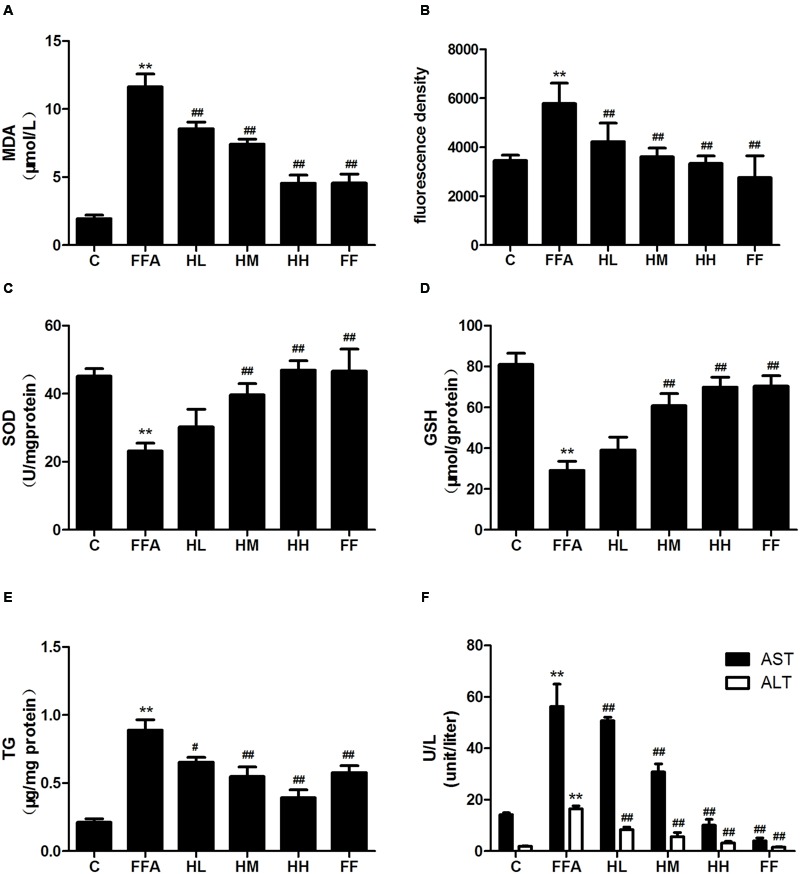
**Effects of Hugan Qingzhi (HQT)-medicated serum on levels of MDA (A)**, ROS **(B)**, SOD **(C)**, GSH **(D)**, TG **(E)**, and AST, ALT **(F)** in free fatty acid (FFA)-stimulated L02 hepatocytes over 24 h. Values are expressed as mean ± SD in each group. ^∗∗^*p* < 0.01 vs. control group; ^#^*p* < 0.05 vs. FFA group; ^##^*p* < 0.01 vs. FFA group.

**FIGURE 2:**
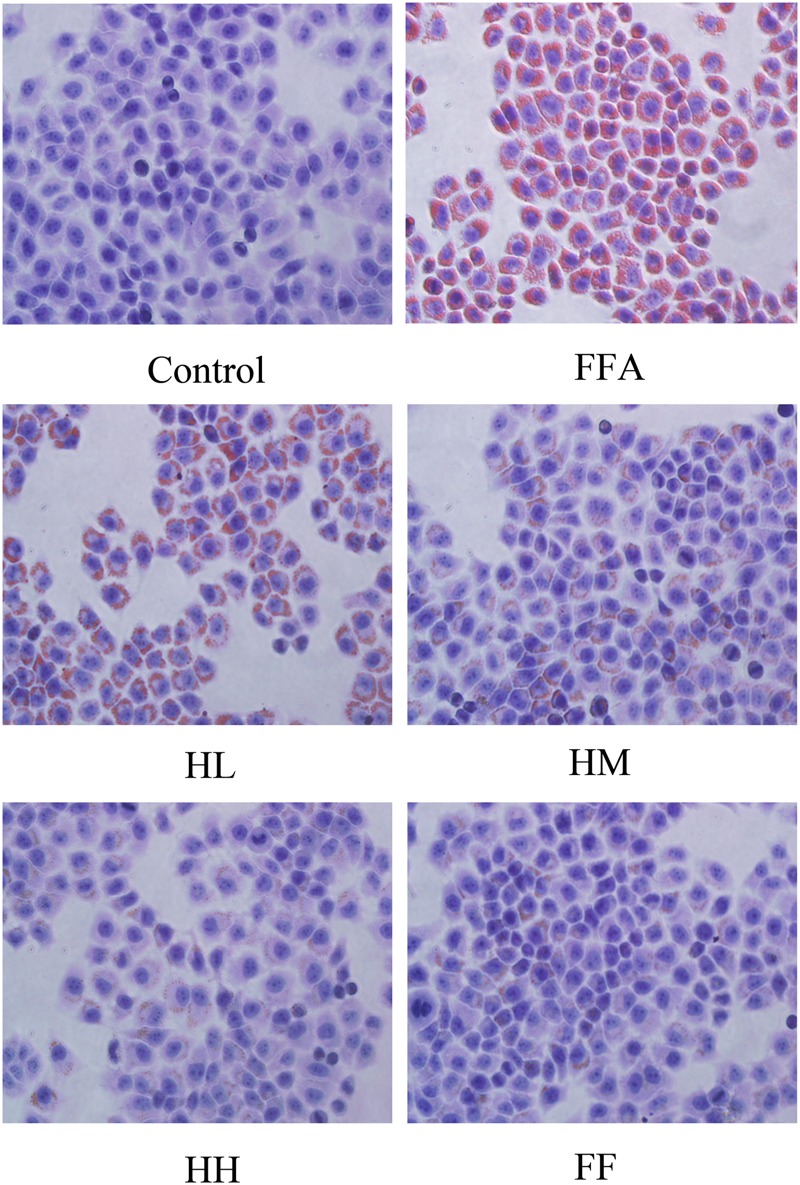
**Effects of HQT-medicated serum on TG examination in FFA-stimulated L02 hepatocytes over 24 h following Oil Red O staining (40 × 10 magnification).** Control, control group; FFA, free fatty acid group; HL, FFA + 10% low dose HQT-medicated serum group; HM, FFA + 10% moderate dose HQT-medicated serum group; HH, FFA + 10% high dose HQT-medicated serum group; FF, FFA + 10% fenofibrate-medicated serum group.

**FIGURE 3:**
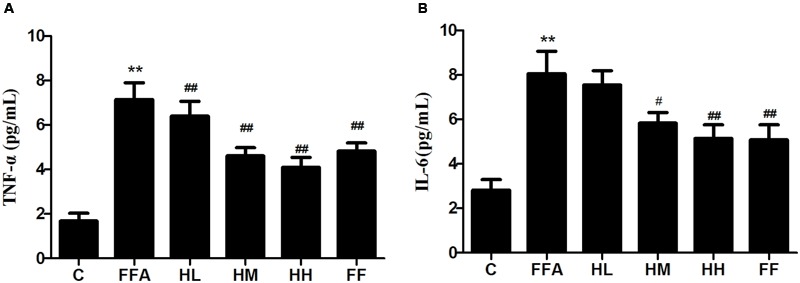
**Effects of HQT-medicated serum on the levels of TNF-α and IL-6 (A,B)** in free fatty acid (FFA)-stimulated L02 hepatocytes over 24 h. Values are expressed as mean ± SD in each group. ^∗∗^*p* < 0.01 vs. control group; ^#^*p* < 0.05 vs. FFA group; ^##^*p* < 0.01 vs. FFA group.

Production of MDA in the culture supernatant, activity of SOD, and intracellular levels of GSH and ROS were all measured in the present study to evaluate whether HQT-medicated serum protects L02 cells injured by FFA. The results showed that production of MDA in the culture supernatant and intracellular ROS stimulated by FFA for 24 h were significantly increased compared with the control group (**Figures 1A,B**). In contrast, SOD activity and GSH levels were reduced (**Figures 1C,D**). In comparison with the FFA group, the HQT-medicated serum group exhibited not only a down-regulation of MDA production and intracellular ROS, but also an up-regulation of SOD activity and GSH levels in a dose-dependent manner. HQT-medicated serum also showed an ability to inhibit FFA-induced intracellular triglyceride (TG) accumulation (**Figure 1E**) in L02 hepatocytes. In the Oil Red O staining assay, treatment of L02 cells with FFA resulted in a considerable increase in TG accumulation, whereas treatment with HQT-medicated serum significantly attenuated TG accumulation (**Figure 2**), a finding that is consistent with those presented in **Figure 1E**. In comparison with the FFA group, AST and ALT levels in the culture supernatant of the HQT-medicated serum treatment group were significantly reduced (**Figure 1F**). In the assay to detect TNF-α and IL-6, both showed a significant increase in FFA-treated cells, compared with the control group, whereas cells treated with HQT-medicated serum showed a marked inhibitory effect (**Figures 3A,B**). These results all suggest that HQT-medicated serum can regulate oxidative stress and inflammatory cytokine production, as well as ameliorate intracellular TG accumulation to achieve an anti-NAFLD effect.

### Quantitative Identification of Differentially Expressed Proteins Using iTRAQ in L02 Cells

**FIGURE 4:**
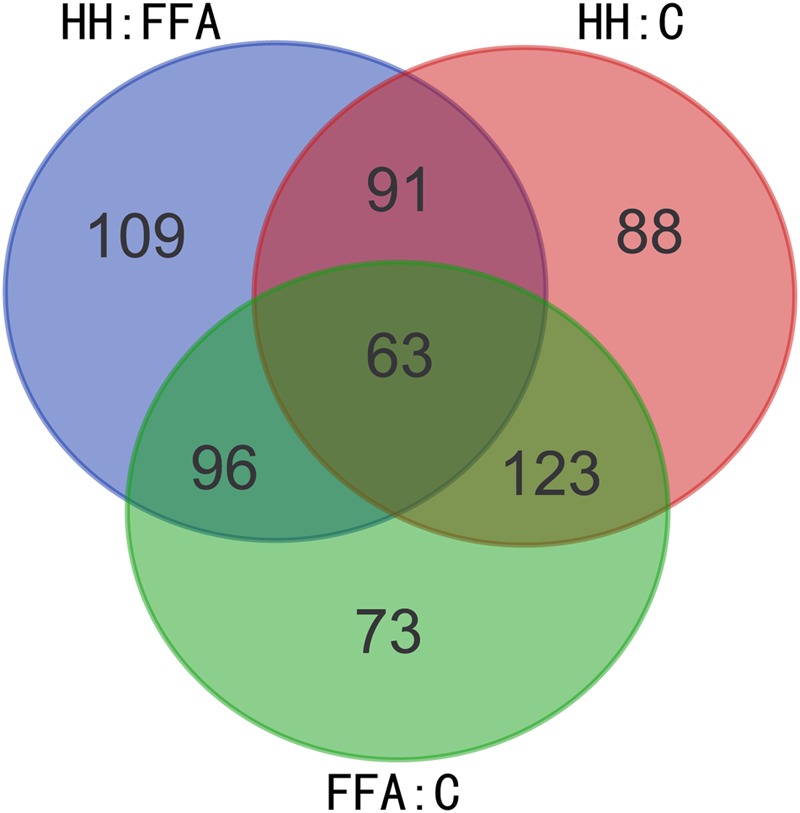
**Venn-diagram showing overlap of all differentially expressed proteins among FFA: C, HH: FFA, and HH: C**.

The protective effects of HQT-medicated serum have been proven on FFA-stimulated L02 cells. However, the molecular mechanisms of HQT-medicated serum on FFA-induced L02 cell damage are still not fully understood. Thus, an iTRAQ-based proteomic method was used to analyze the molecular mechanisms of this process. After merging data from the respective replicates, 4307 proteins were identified, of which, 3586 (83.26%) contained at least two unique peptides. Moreover, 4268, 4269, and 4269 proteins were quantified in FFA:C, HH:C, and HH:FFA, respectively. Further analysis identified that there were 355 (169 up-regulated, 186 down-regulated), 365 (203 up-regulated, 162 down-regulated), and 359 (232 up-regulated, 127 down-regulated) differentially expressed proteins in FFA:C, HH:C, and HH:FFA, respectively (**Table 3** and Supplementary Table S1). The Venn-diagram in **Figure 4** shows the overlap of all differentially expressed proteins among the three groups.

**Table 3:** Number of differentially expressed proteins identified in experiments.

Sample pairs	FFA: C	HH: C	HH: FFA
Quantified	4268	4269	4269
Up-regulated	169	203	232
Down-regulated	186	162	127
Total difference	355	365	359

*C, the control group; FFA, free fatty acid group (1 mM); HH, FFA + 10% high dose HQT-medicated serum group.*

### Bioinformatics Analysis of Differentially Expressed Proteins (DEPs)

**FIGURE 5:**
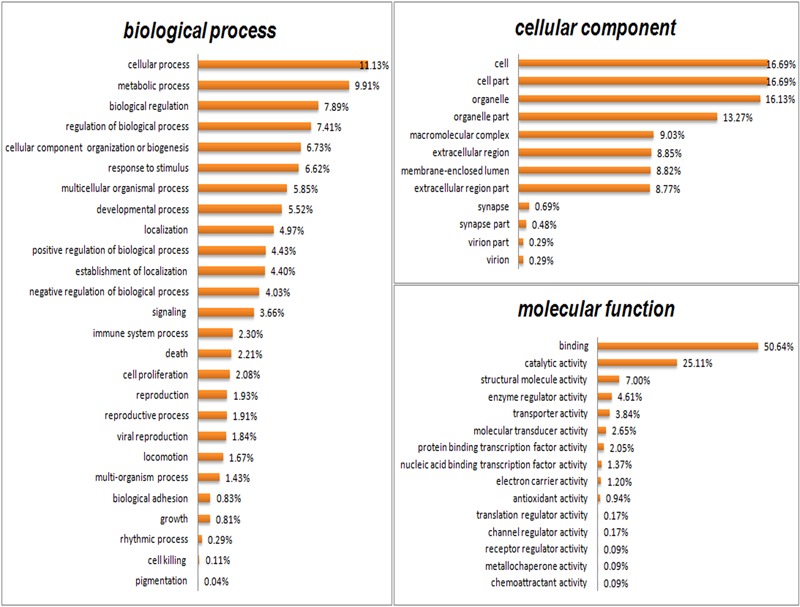
**Gene Ontology (GO) analysis of all differentially expressed proteins.** Proteins were classified according to biological process (BP), cellular component (CC), and molecular function (MF) by Blast2GO software (v4.5 pipeline5).

GO analysis was performed to functionally classify the differentially expressed proteins (DEPs), with the proteins categorized according to biological process (BP), cellular components (CC), and molecular functions (MF) using the Blast2GO software (v4.5 pipeline5). As shown in **Figure 5**, proteins were separated according to their BP into 26 subcategories that included the following: cellular process (11.13%), metabolic process (9.91%), biological regulation (7.89%), regulation of BP (7.41%), CC organization or biogenesis (6.73%), localization (4.97%), and developmental process (5.52%), among others. The eight main CC subcategories were: cell (16.69%), cell part (16.69%), organelle (16.13%), organelle part (13.27%), membrane-enclosed lumen (8.82%), macromolecular complex (9.03%), extracellular region part (8.77%), and extracellular region (8.85%). The four major MF subcategories were: binding (50.64%), catalytic activity (25.11%), enzyme regulator activity (4.61%), and structural molecular activity (7.00%).

The DEPs were further characterized in FFA:C, HH:C, and HH:FFA, respectively, using GO enrichment analysis (Supplementary Figures S1–S3). Several differences were noted in the percentages of some GO terms. For example, only down-regulated proteins showed antioxidant activity in FFA:C. In HH:C, however, both down-regulated and up-regulated proteins showed antioxidant activity. For electron carrier activity, the percentage of up-regulated proteins was higher than that of down-regulated proteins in FFA:C, whereas this phenomenon was reversed in HH:C. These results suggest that HQT-medicated serum protects FFA-induced L02 cell damage by improving antioxidant activity, an action that might be directly or indirectly associated with its actions in the mitochondrial electron transport chain.

To analyze the biological pathways that responded to FFA-induced damage and the effects of HQT-medicated serum in FFA:C, HH:C, and HH:FFA, respectively, all DEPs were further analyzed using the KEGG database. A total of 315, 322, and 313 proteins were categorized in FFA:C (151 up-regulated, 164 down-regulated), HH:C (174 up-regulated, 148 down-regulated), and HH:FFA (197 up-regulated, 116 down-regulated), respectively (Supplementary Table S2). Among the DEPs in FFA:C, HH:C, and HH:FFA, a total of 185, 179, and 187 biological pathways were annotated, respectively (Supplementary Table S3). It was also found that many pathways among the three groups were enriched, including microbial metabolism in diverse environments, fatty acid metabolism, PPAR signaling pathway, and mitogen-activated protein kinase (MAPK) signaling pathways, among others.

### Verification of Differentially Expressed Proteins by Western Blotting

**FIGURE 6:**
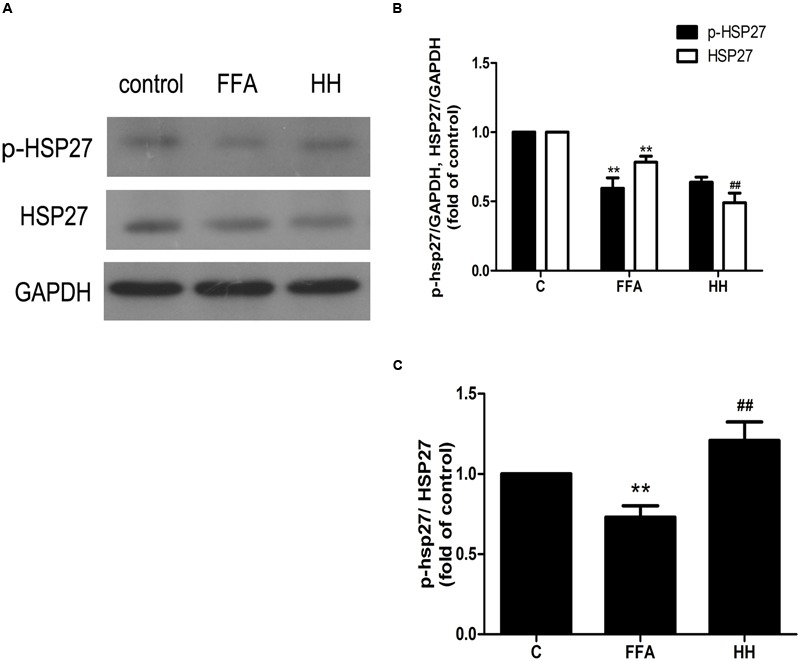
**Effects of HQT-medicated serum on amount of phosphorylated HSP27 and total HSP27 (A,B)** and phosphorylation level of HSP27 **(A,C)** in FFA-stimulated L02 hepatocytes over 24 h. GAPDH was used as a loading control. Values are expressed as mean ± SD in each group. ^∗∗^*p* < 0.01 vs. control group; ^##^*p* < 0.01 vs. FFA group.

**FIGURE 7:**
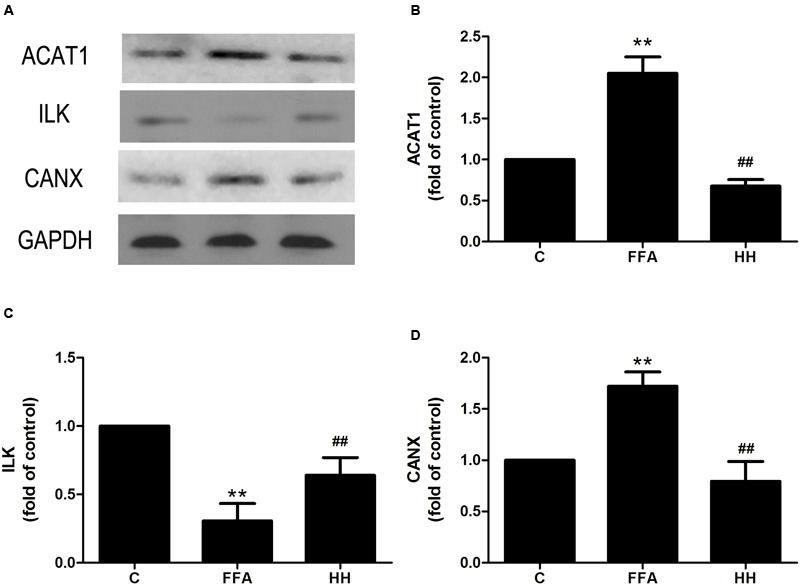
**Effects of HQT-medicated serum on expression of ACAT1 (A,B)**, ILK **(A,C)**, and CANX **(A,D)** expression in FFA-stimulated L02 hepatocytes over 24 h. GAPDH was used as a loading control. Values are expressed as mean ± SD in each group. ^∗∗^*p* < 0.01 vs. control group; ^##^*p* < 0.01 vs. FFA group.

Several iTRAQ-identified proteins were selected for validation by western blotting, including acetyl-CoA acetyltransferase 1 (ACAT1), integrin linked kinase (ILK), heat shock protein 27 (HSP27, also referred to as HSPB1), and calnexin (CANX). These proteins were annotated into four signaling pathways: fatty acid metabolism (ACAT1), PPAR signaling pathway (ILK), MAPK signaling pathway (HSP27), and protein processing in the endoplasmic reticulum (ER; CANX). Results showed that in comparison with the control group, ACAT1 and CANX were up-regulated by FFA treatment alone, whereas ILK and phosphorylated HSP27 were down-regulated. In comparison with the FFA group, expression of these proteins was reversed in the HQT-medicated serum group (**Figures 6, 7**). The results detected by immunoblotting analysis were consistent with those of iTRAQ-identification, indicating that the protective effects of HQT-medicated serum in L02 hepatocyte damage induced by FFA are associated with expression and function of the respective proteins.

### Role of HSP27 in FFA-Induced L02 Cell Damage and Effects of HQT-Medicated Serum

Among the four validation proteins, the expression level of HSP27 in the present study was remarkable. HSP27 is a multidimensional protein with several functions, which is usually activated in response to cellular stress, including oxidative stress and chemical stress. In the present study, FFA treatment over 24 h resulted in a decline in HSP27 activity, as determined by immunoblotting analysis with an antibody against the phosphorylated form of HSP27. These findings are consistent with those of iTRAQ-identification. In contrast, HQT-medicated serum caused an increase in HSP27 activity in LO2 cells. Thus, to investigate further the activity of HSP27 during FFA treatment, the expression levels of phospho-HSP27 and total HSP27 were detected following FFA treatment at different intervals.

**FIGURE 8:**
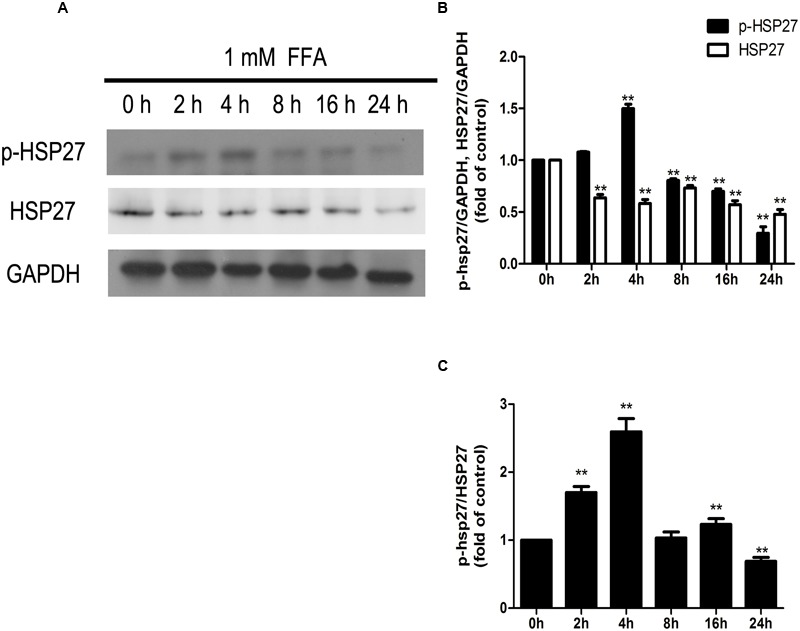
**Dynamic changes in the amounts of phosphorylated HSP27 and total HSP27 (A,B)**, and phosphorylation level of HSP27 **(A,C)** during the process of FFA treatment. GAPDH was used as a loading control. Values are expressed as mean ± SD in each group. ^∗∗^*p* < 0.01 vs. 0 h group.

As shown in **Figure 8**, expression of phosphorylated HSP27 was increased following FFA treatment in a time-dependent manner for 4 h, and later declined as treatment time increased. Moreover, total HSP27 expression showed a consistent decline with time, as FFA treatment progressed.

**FIGURE 9:**
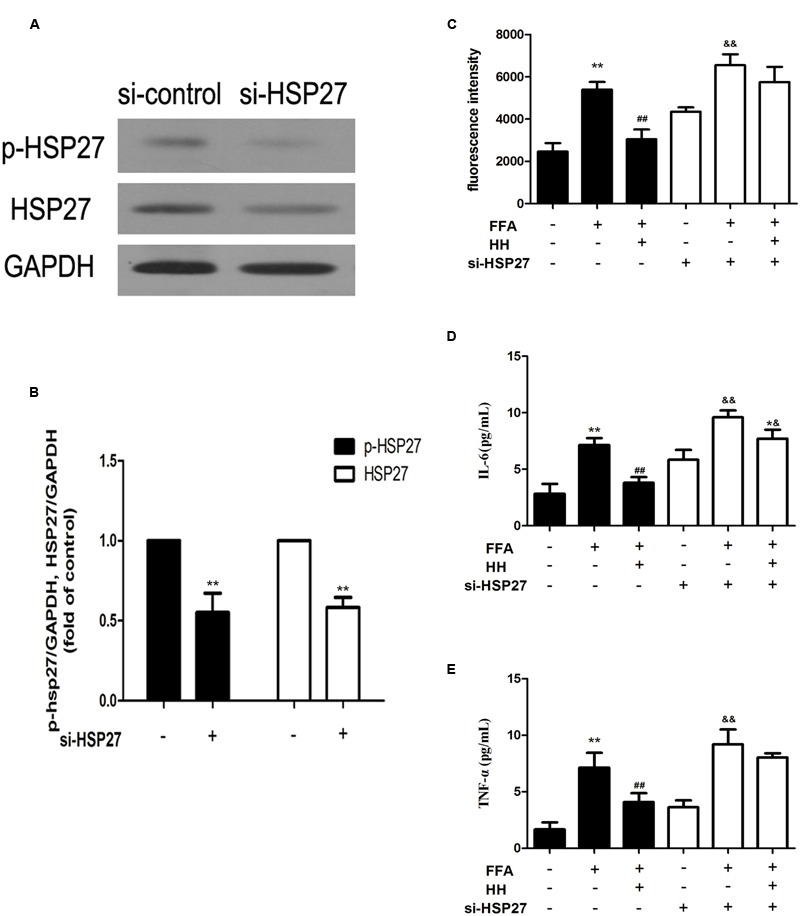
**Expression of total and phosphorylated HSP27 after treatment with HSP27 siRNA (A,B)**. Effects of HQT-medicated serum on levels of ROS, TNF-α, and IL-6 induced by free fatty acids (FFA) in normal L02 cells and HSP27 knockdown L02 cells **(C–E)**. GAPDH was used as a loading control. Values are expressed as mean ± SD in each group. ^∗∗^*p* < 0.01 vs. control group; ^##^*p* < 0.01 vs. FFA group; ^&&^*p* < 0.01 vs. si-HSP27 group; ^∗&^*p* < 0.05 vs. FFA + si-HSP27 group.

To determine the involvement of HSP27 in FFA-stimulated L02 cells and the effects of HQT-medicated serum, specific siRNA against HSP27 was applied to L02 cells. As shown in **Figures 9A,B**, HSP27 siRNA significantly knocked down total and phosphorylated HSP27 expression. Levels of ROS, IL-6, and TNF-α were again evaluated after HSP27 was knocked down, as shown in **Figures 9C–E**. In normal cells, HQT-medicated serum significantly attenuated FFA-induced ROS, IL-6, and TNF-α production compared with FFA treatment alone. Nevertheless, in HSP27 knocked down cells, the attenuated effects of HQT-medicated serum on ROS (*p* = 0.1945), IL-6 (*p* = 0.03), and TNF-α (*p* = 0.28) production induced by FFA were weakened, compared with the FFA group. When combined, these data suggest that activation of HSP27 inhibits FFA-induced ROS, IL-6, and TNF-α production, and that the protective effects of HQT-medicated serum in FFA-induced L02 cell damage are associated with its regulation of HSP27.

## Discussion

A previous study demonstrated that the anti-hepatic steatosis effect of HQT in L02 hepatocytes inhibits SREBP-1 expression and increases CPT-1 and ACOX-1 expression via activation of the AMPK and PPAR-α pathways (Yin et al., 2014). Although that study provided evidence of the anti-hepatic steatosis and antioxidant effects in FFA-induced L02 hepatocyte damage, the molecular mechanism underlying the protective effects of HQT remained largely unknown. As a follow-up, the present study employed quantitative proteomics to elucidate the underlying mechanisms of HQT in FFA-induced L02 hepatocyte damage. The results of biochemical assays (including AST, ALT, and MDA, etc.) further confirmed the protective effect of HQT in FFA-induced L02 hepatocyte damage, consistent with the results of the previous study (Yin et al., 2014). Furthermore, proteomic analysis revealed that HQT protects against L02 hepatocyte injury induced by FFA, and is associated with protein processing in the ER, fatty acid metabolism, and the PPAR and MAPK signaling pathways.

Accumulation of intrahepatic TG (hepatic steatosis) is one of the most remarkable and characteristic changes associated with NAFLD, and usually occurs when the rate of fatty acid input is greater than that of the output. There are two aspects to fatty acid input: *de novo* synthesis and uptake from food. Output entails fatty acid oxidation and esterification to produce TG. TG are either incorporated into lipoproteins or stored as lipid droplets (Kohjima et al., 2007). Therefore, proteins involved in fatty acid metabolism are likely to play an important role in processes associated with NAFLD. In the present study, ACAT1 was up-regulated in the FFA group compared with the control group, and was inhibited by HQT-medicated serum. ACAT1 is responsible for cholesteryl ester (CE) synthesis. In most cell types, CE mainly stored as cytoplasmic lipid droplets (Chang et al., 1997). On the other hand, the up-regulation of ACAT1 is noticeable in NAFLD, which is accompanied by over-production of cholesterol (Nakamuta et al., 2009). Over production of cholesterol can lead to increased levels of oxysterols, activation of LXR-α and SREBP-1, and enhance fatty acid synthesis (Nakamuta et al., 2009). Therefore, ACAT1 may be a potential marker of NAFLD, suggesting that the anti-hepatic steatosis effect of HQT is associated with regulation of ACAT1.

Accumulation of TG within hepatocytes caused by abnormalities in lipid metabolism and disposition is the “first hit” in development of NAFLD (Basaranoglu et al., 2013). An additional stressor in the incidence and development of NAFLD is sometimes referred to as a “second hit,” and can include oxidative stress and the inflammatory response (Basaranoglu et al., 2013). Several pathways, including the MAPK pathway, participate in the progression of oxidative stress and the inflammatory response. The present showed that in comparison with the control, HSP27 is inactivated by FFA treatment over 24 h, and activated by HQT, when compared with the FFA group. Moreover, the expression of phosphorylated HSP27 was increased by FFA treatment in a time-dependent manner for 4 h and declined thereafter as FFA treatment progressed, indicating that levels of phosphorylated HSP27 changed dynamically during FFA treatment. It is speculated that FFA induces a transient increase in phosphorylated HSP27 in L02 hepatocytes. Furthermore, sustained exposure to metabolic stress might reduce levels of phosphorylated HSP27, suggesting that L02 hepatocytes failed to exhibit a robust physiological defense to prolonged cellular stress. Other studies have reported that HSP27 is a reversibly phosphorylated protein which is controlled by protein kinases and phosphatases. Protein kinases can phosphorylate HSP27 in response to cellular stress, including oxidative stress and chemical stress, and thereby demonstrate protective effects under stressful conditions (Ferns et al., 2006; Thomas et al., 2008). On the other hand, phosphorylation of HSP27 is also controlled by phosphatases that effect dephosphorylation of HSP27 (Berrou and Bryckaert, 2009; Kostenko and Moens, 2009). Moreover, one clinical study reported that HSP27 is down-regulated in ballooned hepatocytes of patients with NASH, indicating that the *in vitro* model of NAFLD established in the present study is very similar to that of clinical NASH with ballooning degeneration (Sookoian et al., 2016). Furthermore, using HSP27-specific siRNA, it was discovered that the inhibition effects of HQT on ROS generation and production of IL-6 and TNF-α in FFA-treated L02 hepatocytes are involved in recovery, following activation of HSP27. These findings indicate that HSP27 plays an important role in progression of NAFLD, and HQT attenuates FFA-induced oxidative stress and the inflammatory response by promoting recovery, after the activation of HSP27.

A growing body of evidence has shown that hepatocyte death plays a pivotal role in transition of NAFLD to NASH. Abnormal accumulation of fatty acids in the liver stimulates ROS generation, which can directly induce DNA/protein damage and promote hepatic failure via activation of stress-related signaling pathways, including the MAPK and PPAR pathways (Hardwick et al., 2009). In the present study, ILK was involved in the PPAR pathway. Moreover, HQT can reverse ILK expression induced by FFA to normal levels, because ILK activation in mammalian cells results in suppression of apoptosis and anoikis (Wu and Dedhar, 2001; Li et al., 2016). Thus, HQT might inhibit FFA-induced L02 hepatocyte damage by recovering ILK expression.

The ER is the cellular site of Ca^2+^ storage for synthesis, folding, and maturation of most secreted and *trans*-membrane proteins. Disturbed hepatic cholesterol homeostasis and free cholesterol accumulation in the liver results in increased oxidative stress, leading to ER stress that is closely associated with the development of NAFLD and entails fat accumulation, insulin resistance, inflammation, and apoptosis among other conditions (Samali et al., 2010; Bozaykut et al., 2016). Moreover, CANX, an ER-induced protein, is reportedly up-regulated during ER stress, indicating its importance as a marker of ER stress (He et al., 2016; Ryan et al., 2016). In the present study, HQT inhibited FFA-induced CANX over-expression that was annotated in protein processing in the ER. This finding suggests that the protective effect of HQT is associated with regulation of ER stress.

In the proteomics data, KEGG enrichment analysis revealed that among all DEPs, a total of 31, 21, and 35 proteins from FFA:C, HH:FFA, and HH:C, respectively, were enriched in microbial metabolism in diverse environments. This indicates that development of NAFLD is closely related to microbial metabolism in the host. However, no follow-up evaluations, such as determination of the diversity of gut microbiota, were performed in the present study to confirm these results. A growing body of evidence suggests that gut microbiota act as regulators of energy homeostasis and fat deposition, thereby implicating them in the development of obesity and metabolic disease (Cope et al., 2000; Nair et al., 2001; Rao, 2008). Moreover, changes in secondary metabolites, such as ethanol, ammonia, phenols, and lipopolysaccharide (LPS), induced by alterations in gut microbiota communities can exert the effects of endotoxins which can stimulate the incidence of oxidative stress and inflammation (Aqel and DiBaise, 2015). Therefore, gut microbiota communities could have a crucial role in the progression of NAFLD and could be associated with the protective effect of HQT.

## Conclusion

Using the combined strategy of iTRAQ with LC-MS/MS and subsequent investigation, this study confirmed that HQT has excellent protective effects in FFA-induced L02 hepatocyte injury. These effects were associated with the reduction of lipid accumulation, oxidative stress, and inflammation, and inhibition of ER stress and hepatocyte death. Subsequent validation experiments proved that ACAT1, HSP27, ILK, and CANX might be associated with the protective effect of HQT in the FFA-induced L02 hepatocyte injury model. Moreover, bioinformatic analysis of the DEPs results showed that microbial metabolism in diverse environments was strongly associated with the protective effect of HQT in FFA-induced L02 hepatocytes, suggesting a key role for the gut microbiota in progression of NAFLD. Consequently, the present study not only expands our understanding of the pathogenesis of NAFLD, and provides new insights for its treatment, but further elucidates the underlying mechanism of the effects of HQT in NAFLD treatment. Further studies are needed to clarify the role of DEPs in the effects of HQT in NAFLD treatment, in particular in primary cells or even *in vivo* study.

## Author Contributions

FX and XY are responsible for the experiment operation. WT, CX, and MY are responsible for assisting the experimental design and experiment operation. BZ guided the design of the experiment and is responsible for modification of the paper.

## Conflict of Interest Statement

The authors declare that the research was conducted in the absence of any commercial or financial relationships that could be construed as a potential conflict of interest.

## Footnotes

1 http://www.uniprot.org/

2 http://www.genome.ad.jp/kegg/pathway.html

## Abbreviations

DEPs: differentially expressed proteins
FFA: free fatty acid
GO: Gene Ontology
HQT: Hugan Qingzhi
iTRAQ: isobaric tags for relative and absolute quantification
KEGG: Kyoto Encyclopedia of Gene and Genomes
