# Supplementary material for: Isobaric Tags for Relative and Absolute Quantitation (iTRAQ)-Based Proteomic Analysis of Hugan Qingzhi and Its Protective Properties against Free Fatty Acid-Induced L02 Hepatocyte Injury
Source: Front Pharmacol. 2017 Feb 28;8:99. doi: 10.3389/fphar.2017.00099 (PMC5329039; doi:10.3389/fphar.2017.00099)
Supplement: Supplementary file 1 [file Data_Sheet_1.DOCX]

Supplementary Material

**Isobaric tags for relative and absolute quantitation (iTRAQ) -based proteomic analysis of Hugan Qingzhi and its protective properties against free fatty acid-induced L02 hepatocyte injury**

Fan Xia *, Xiaorui Yao, Waijiao Tang, Chunxin, Xiao, Miaoting, Yang, Benjie Zhou

*** Correspondence:** Benjie Zhou: zhoubj163@163.com

# Supplementary Figures and Tables

## Supplementary Figures

**
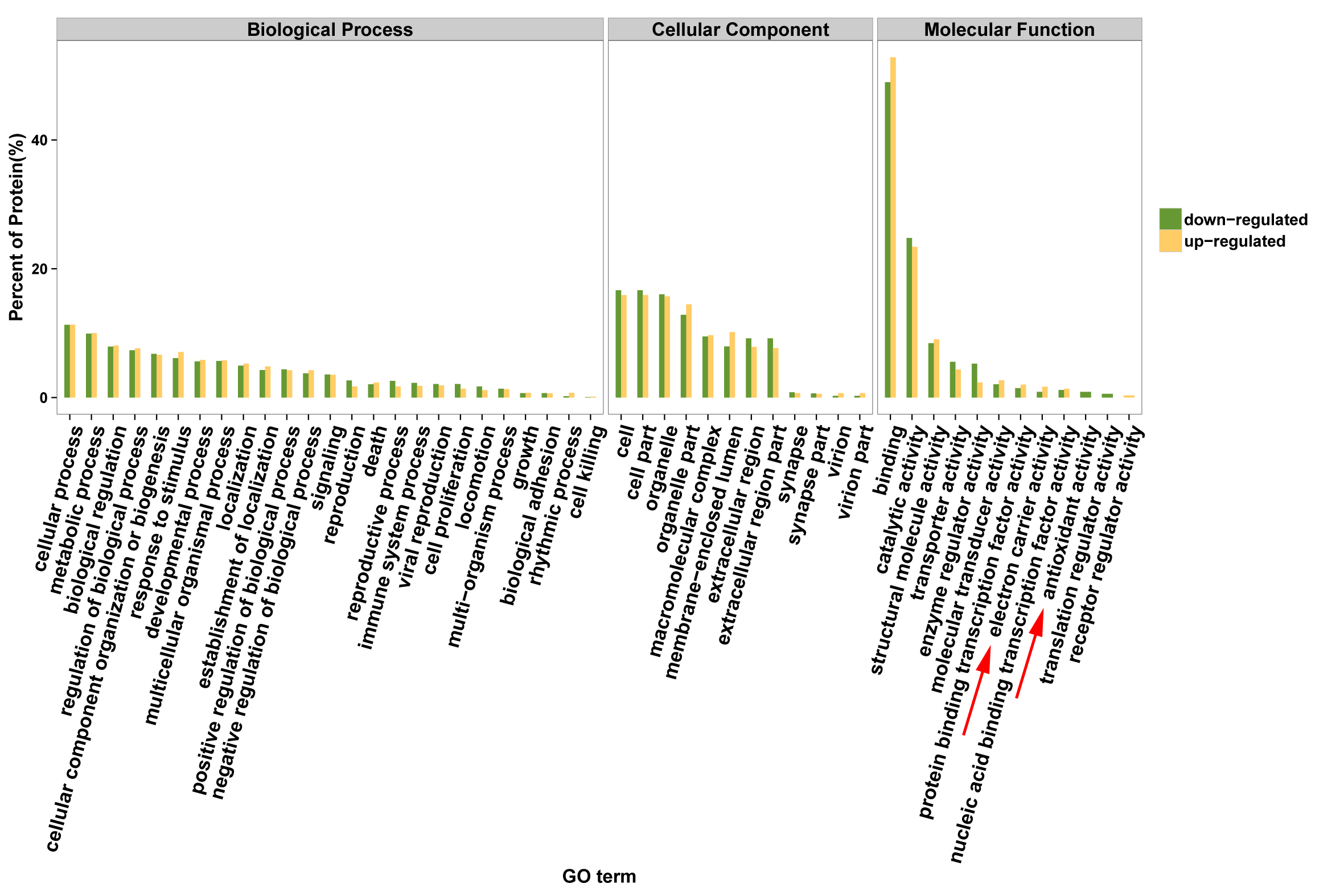
**

**Supplementary Figure 1**. GO enrichment analysis of differentially expressed proteins in FFA:C. Proteins were classified according to biological process (BP), cellular component (CC), and molecular function (MF).


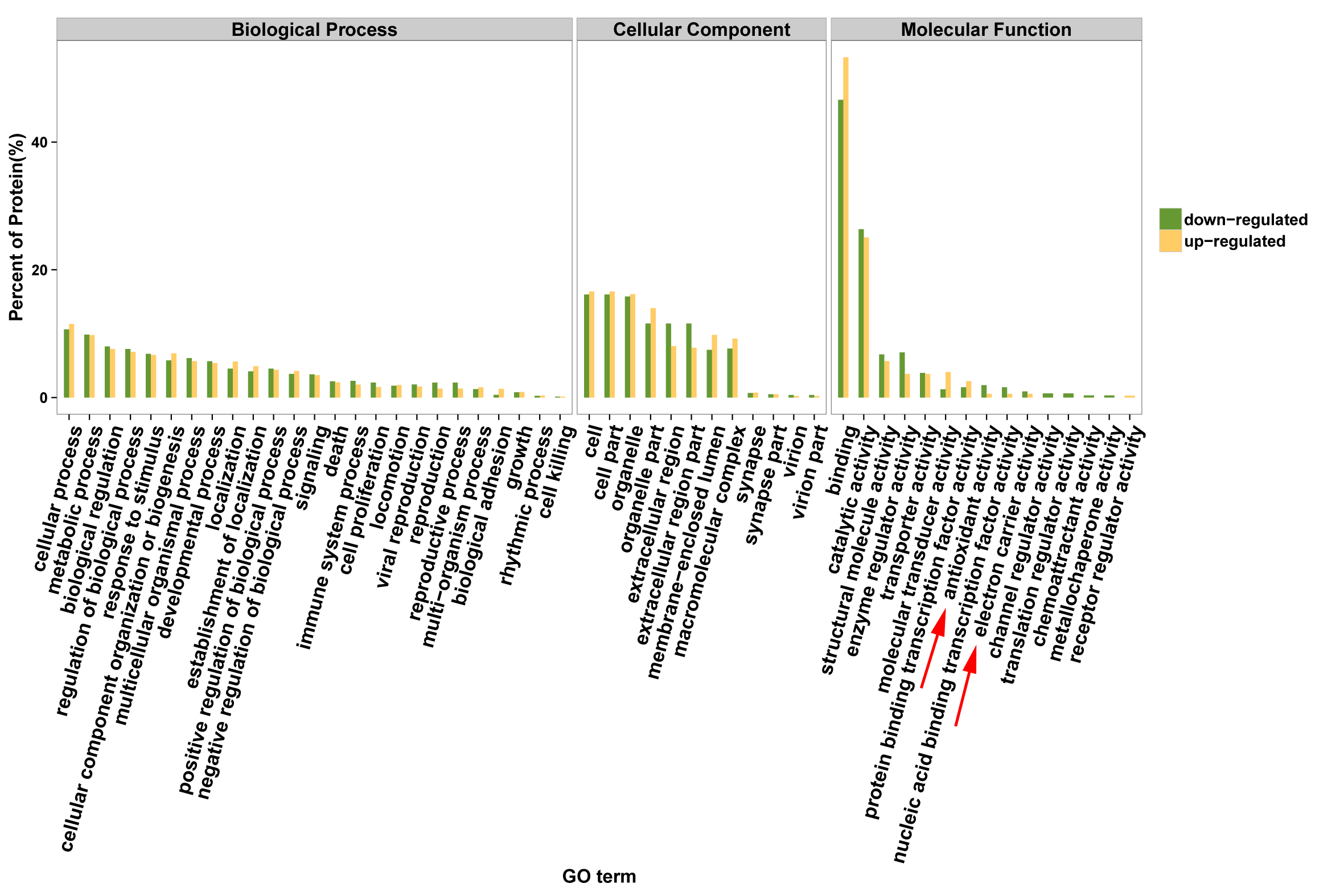


**Supplementary Figure 2**. GO enrichment analysis of differentially expressed proteins in HH:C. Proteins were classified according to biological process (BP), cellular component (CC), and molecular function (MF).


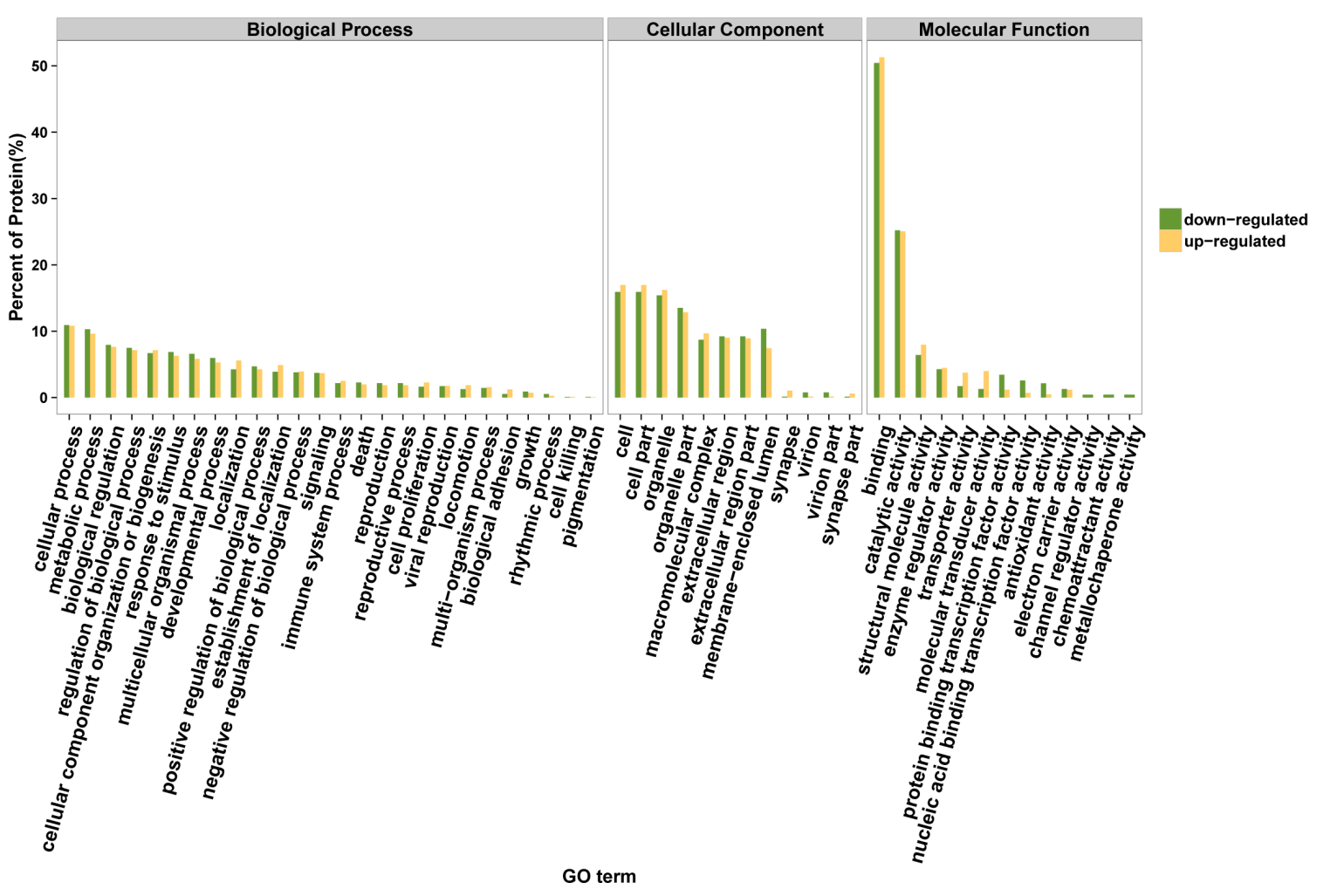


**Supplementary Figure 3**. GO enrichment analysis of differentially expressed proteins in HH:FFA. Proteins were classified according to biological process (BP), cellular component (CC), and molecular function (MF).

## Supplementary Tables

**Supplementary Table S1**. Data of DEPs in FFA: C, HH: FFA, and HH: C, respectively.

**Supplementary Table S2**. Lists of DEPs in KEGG enrichment analysis.

**Supplementary Table S3**.Number of DEPs enriched in different pathways in FFA: C, HH: FFA, and HH: C, respectively.
